# Perception of Stakeholders for Meat Qualities among Value Chain Actors in Ethiopia

**DOI:** 10.1155/2022/1247459

**Published:** 2022-11-21

**Authors:** Birmaduma Gadisa Muleta

**Affiliations:** Oromia Agricultural Research Institute, Bako Agricultural Research Center, Bako, Ethiopia

## Abstract

Perception is the knowledge of an individual/group gained from experience and impressions of the ideal situation. Ethiopian livestock marketing has a different stage and large actors. However, in the flow chain, there is less communication. Because they only perceive for their benefit rather than care for a quality product. This happened because the production mapping was not understood among them. The majority of producers fetch too old animals after being culled from production and those that might be abnormal due to disease and chronic stress. Traders transport mixed animals through nondedicated vehicles and long trekking without feeding and watering. Abattoir men worked with poor facilities. They perceived that the time stay animals in Lairage, breeding, bleeding, and carcass handling is the major problem in meat quality in Ethiopia. The slaughtering has been conducted in brutal ways of stunning using either a hammer or knife at the atlanto-occipital space of the animal on the floor side by side. The majority of butchers in Ethiopia are located on the main road for their products to be easily displayed to clients, and they hang meat on the open shelf without packing, which exposes the product to aerobic spoilage by bacteria and yeasts. Traders/brokers are promoting the product based on the commission they earn rather than the quality and health of the animals. However, as a principle, each actor has a responsibility to manage risk as they benefit socially and economically from firms. Government entities should play an important role in shaping actors' perceptions and understanding of biosecurity measures. Mainly, the interventions should focus on improving business models and technological adoption. This model is used for improving vertical relationships among operational actors, horizontal relationships with logistics providers, and market promotion measures to attract foreign direct investors and importers, transforming traditional practices of animal husbandry into commercial ones. Because a key activity for each value chain actor is availing of the final product safely at the right place and time. The review was designed to convey information to enhance the linkage between meat value chain actors and optimize management skills in Ethiopia.

## 1. Introduction

Perception is the way an individual/group understands something based on his/her/their experience, facts, stories, impressions, or ideal situation [1–3]. A firm's value chain is an interdependent network of activities between actors [4]. In Ethiopia, the agricultural sector is a cornerstone of the economic and social life of the people. But, the low output and input is outstanding due to the rain-fed farming system being overwhelmed. Livestock is an integral part of agriculture in Ethiopia. However, the contribution of live animals and their products has not been fully exploited in Ethiopia due to the fact that most of the production system is subsistence-oriented [5–7].

A central issue in food marketing is the value chain. Ethiopian livestock marketing is characterized by poor market infrastructure and poor technical knowledge of value chain actors, inadequate market information, and poor linkages among actors [8]. This is because unresponsive and engaging multiattitude business linkages were severe problems in the livestock value chain in Ethiopia [5, 9, 10]. Every value chain actors have a responsibility to manage risk as they benefit socially and economically from activities [11]. However, in the Ethiopian live animal and meat marketing chain, individual actors perceive only for their own benefit because the production mapping is not understood between actors [4, 12].

The majority of producers fetch too old animals after being culled from production [13] and those that might be abnormal due to disease and stress [12]. Traders transport animals through nondedicated vehicles and long trekking without feeding and watering [6, 7, 14]. Abattoirs in Ethiopia have poor facilities [7]. Butcher men in Ethiopia hanged meat on the open shelf or unpacked from dust, flies, mold, and poor hygiene [15]. Usually, the butchers in Ethiopia are located on the main road for their product to be easily displayed to clients, which exposed the product to aerobic spoilage by bacteria and yeasts [16]. This states that street foods are unsafe, mainly because of the environment, which might be bus terminals, industrial sites, marketplaces, and other street corners. The butchers might not sterilize their equipment before and after use [13]. However, there is a truncated awareness of the economic benefits of adopting biosecurity measures and their principle costs [3]. According to [17]reports Ethiopia lost about 28.45 USD from only every infected slaughtered cattle in (year). Hence, the sectors need professional and policy interventions to transport reliable and sustainable live animals and their products across market signals from the producer to the consumer [6].

The mapping is denoted as a functional and institutional analysis to provide an overview of all chain actors and the type of interaction between them [18]. Government entities play an important role in shaping actors' perceptions and understanding of biosecurity measures. Mainly, the interventions should focus on improving business models. This model is for improving vertical relationships between operational actors, horizontal relationships with logistics providers, market promotion measures to attract foreign direct investors and importers, and transforming traditional practices of animal husbandry into commercial ones. Transporting animals by using dedicated cargo than using goods vehicles can minimize the stress. The slaughtering in Ethiopia is conducted by fewer facilities; hence, support by HACCP and ISO 22000 certification in an abattoir is mandatory [19, 20].

Most of the time, resource-poor actors cannot justify and afford to invest in innovations and measures that they regard as vague and generating no tangible benefits [21]. It is vital to understand that adoption is driven by access to capital, education levels, training, less cost, ease of implementation, legal environment, and benefits [22]. Moreover, value chain actors have seen the direct benefits of adopting biosecurity measures; hence, they are reluctant to voluntarily invest. To date, developing countries have not sufficiently invested in research institutions, policies, and infrastructure in informal value chains [23]. These authors have realized that there are several challenges related to the noncooperation of stakeholders regarding meat quality in the cases of Ethiopia. Therefore, this mini-review analyses the attitude of stakeholder groups toward meat quality across the livestock value chain outlined in Ethiopia and suggests a prospective prosperous direction for the sector.

## 2. Overview of the Live Animals and Meat Value Chain in Ethiopia

**Figure 1:**
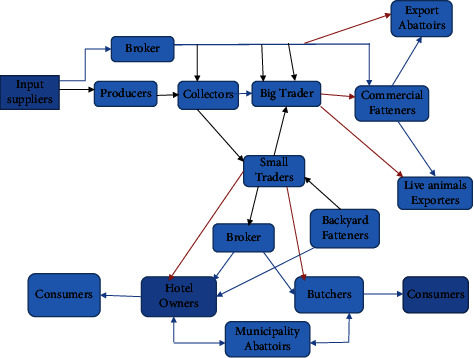
Live animals and the meat value chain in Ethiopia. Source: AACCSA [14].

Most animals are transported at least once in their lifespan from the farm to auction centers [14, 24]. Ethiopian livestock marketing is characterized by poor market infrastructure, inadequate market information, and poor linkages among value chain actors [8]. This poor value chain development could eat into the profitability of the actors. This is because a key activity for each value chain actor is availing the final product at the right place and time. The one threat to the sustainability of the sector was the involvement of enormous actors (input suppliers, producers (backyards and feedlots), traders (collectors, small and large traders, exporters), abattoirs (backyard, municipality, and export), brokers (in every stage), meat traders (butchers, restaurants, hotels, and supermarkets) and end consumers and multistaged service providers), which is well-presented on the map (Figure 1). However, brokers have more communication than other actors (Figure 1). The policy designers, capacity building for actors, and building infrastructure to bring quality along the value chain [5, 8]. Sustainable access to raw materials has been needed for livestock producers for sustainable and higher meat quality source animal supply to market, higher profitability and healthier across the chain.

### 2.1. Livestock Resources and Economic Contribution to Ethiopia

**Figure 2:**
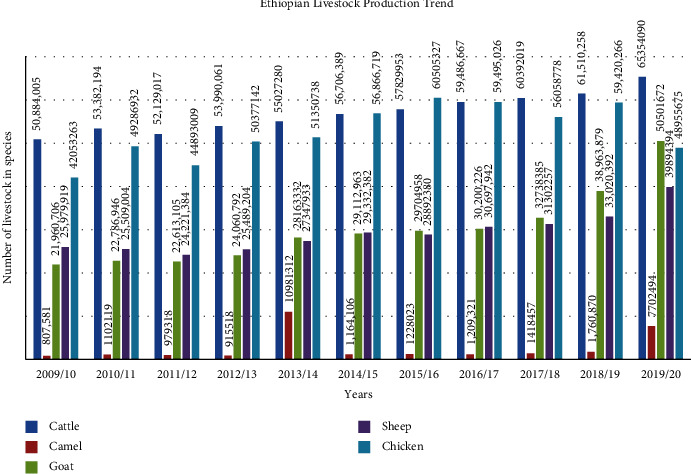
National livestock population statistics indicating promising trends. Source: CSA (2009–2019).

Ethiopia has about 27 cattle, 9 sheep, 12 goat, 10 poultry, and 2 camel breeds [25], and the trend number of ten years for each species is presented in Figure 2. The rapid increments in livestock number and product demand in the world represent a great opportunity for livestock resource-rich Ethiopia. In contrast, the quality and quantity of being utilized and exported are not compared to the stock. The prediction of livestock population growth is expected to be higher (20%) than that of the human population (2%) in the period from 2015 to 2025 [5]. According to the central statistics agency (CSA, 2010–2020), Ethiopian livestock trends indicated that the number of all species is increasing from year to year, as shown in Figure 2. However, the per capita meat consumption remains constant (8.5 kg/year), and Ethiopia imports a significant amount of meat from the USA, UAE, China, Italy, the Netherlands, and South Africa. One of the main reasons given by meat-importing firms in Ethiopia is the unavailability of higher-quality meat in domestic markets [9, 26, 27].

Hence, these controversial reports result in a big assignment for the sector, as deep investigations are needed on why product consumption has been anticipated for years in Ethiopia. Up until now, livestock products have played a crucial role in the development of the nation's economy. Having geographic proximity to the Middle Eastern markets gave a comparative advantage for the quickest delivery time of fresh meat or meat products. In 2015/16, the country secured about 667,005 USD from live animal exports. In this export performance, the country has earned 58.89 million USD, of which about 42.72 million USD was from cattle, 6.57 million USD from camels, and 9.6 million USD from sheep and goats [5].

### 2.2. Livestock Market Stage in Ethiopia

Marketing is an important component of the livestock production systems in Ethiopia. Well-organized livestock marketing plays a crucial role in enhancing the contributions to the well-being of rural communities [28, 29]. Ethiopian livestock marketing has a different stage. However, it is a personalized business with irregular buyers and sellers and with several brokers [5]. Only 20% of live animal exports go through official channels, while the remaining 80% are traded informally [10]. Hence, smallholder farmers face many challenges in livestock marketing, including weak bargaining power, a lack of market information, high transaction costs, limited physical access to markets, poor infrastructure, unorganized markets, and weak institutional support systems [29]. In contrast, virtually all of Ethiopia's red meat exports pass through formal channels due to the high degree of regulation in importing countries [30–32].

#### 2.2.1. Farm Gate Market

Most farmers and rural traders are included in this stage. Rural traders (collectors) come to farmers' houses to estimate the price of the animal. This might occur without the producer looking for it. Mostly, rural traders serve as an agent for small and large traders as well as local butchers, keeping a commission from the sale of the animals. Fixation of prices by local traders on the spot and at the roadside is the major problem with this market. It is difficult to change the price at the primary market since they inform all traders and create a communication gap between buyers and sellers. Usually, rural traders/brokers promote the product based on the commission they earn rather than the quality and health of the animals.

#### 2.2.2. Local (Primary) Market

Pastoralists and highland livestock owners are typically selling their animals to small traders and producers in the primary market. This market is where fewer than 500 head of animals are marketed in a week [33]. There are no facilities for weighting, watering, or feeding, and sometimes there is no fencing. Producers sell animals directly to small-scale traders and sometimes to local butchers and consumers [31]. Usually, this type of market animal is less accustomed to humans at an early stage of their lives; therefore, fear causes novelty and mixed with an unfamiliar animal which behaviors stress [24]. Since the production system is nonmarket-oriented and less market information is available in the area, the animal might be returned to the farm with disease and stress [6, 12, 29].

#### 2.2.3. Secondary Markets

This is the marketplace where small traders and farmers (sellers), big traders, and butcheries (buyers) meet to exchange values in a regional market center. Traders and butchers from terminal markets come to buy animals, which are mostly held at the zone and sometimes at district level. Mostly about 500–1000 heads of different livestock species have been held per week. Farm gate, primary, and secondary markets are the main source of feedlot farms in Ethiopia [34].

#### 2.2.4. Terminal Markets

This is the marketplace where meat animals are slaughtered and displayed for sale in the principal cities. Big traders and butchers are overwhelmed by this market. This market is held in principal towns like *Addis Ababa, Adama, Hawasa, Dire Dawa, Bahar Dar, Mekelle,* and similar big cities in the country. Mostly, more than 1,000 heads of different livestock species have been held in a week [10, 30]. In the terminal market, different breed types are accessed, but the different breeds have different demands from different stakeholders [6].

#### 2.2.5. Abroad Market

Ethiopia has good opportunities to export live animals and their products to Arab countries and South African countries [35, 36]. Annually, over 2.3 million live animals, cattle, sheep, and goat, are exported both formally and informally [31]. This has a comparative advantage in terms of geographic proximity to the Middle East markets, with the potential for the quickest delivery time of fresh meat or meat products [37]. Ethiopia has high-ranked animal breeds such as sheep (Horro, Bonga, Washer, and Arsi-Bale), goats (long-eared Somali, short-eared Somali, Woyito-Guji, and Afar), and cattle (Borana, Fogera, and Horro) [36]. However, exporting live animals from Ethiopia is periodically interrupted due to the bans imposed by importing countries. The main causes of bans on meat exporters are disease outbreaks and unsatisfactory product quality [31]. Hence, Ethiopia should be designing a proper breeding strategy, for improved feeding, health services, product safety, product promotion, and excellent relationships among value chain actors to penetrate the global market like the USA, Japan, and Europe, boost its foreign currency earnings, and promote national products.

In general, there has historically not been a reliable, sustained relationship in the livestock market stage in Ethiopia. Most relationships are casual and change often to suit the situation and the actors [37]. The other challenges of the live animal and meat marketing system in Ethiopia are the absence of an effective grading system, absence of market information, absence of promotional efforts, sustainable supply problems, transport problems, prevalence of diseases, illegal export, inadequacy of infrastructure, competition, repeated bans, and inadequate port facilities [38]. Therefore, repeated bans were reported on the export livestock market in Ethiopia [31]. To harness the potential of the livestock sector, the government has been working on the improvement of some species, ensuring an adequate market supply of quality live animals, increasing the number of export standard abattoirs, engaging professionals in consultation, and introducing the latest technologies to the sector [14, 39].

### 2.3. The Main Operational Actor and Their Function in the Meat Value Chain Map

Value chain actors refer to those individuals or entities that engage in a transaction and products from inception to end-use through explicit negotiation and partner selection [40]. Operational actors are those actors who are doing the actual business from input provision, production, collection, processing, trading, and consumption [14]. However, in the flow chain, there is biased communication among livestock value chain actors in Ethiopia because they only perceive for their own benefit rather than caring for the quality products in their hands for the next firm actors [4, 6].

Therefore, needs intervention for livestock market signals from the producers to the consumers. Because only a little information is available on the relationship between animal behavior characteristics, stock person attitudes across value chain signals in Ethiopia towards handling, behavioral responses before slaughter, and graded meat quality.

In 2016/17, Ethiopia produced 46,120 tons of meat and exported only 19,104.7 tons (41.4%) of meat [41]. Although Ethiopia has the tenth largest livestock population in the world, the production of meat is still low and contributes to only about 0.2% of the world's total meat production, of which most is limited to sheep and goat meat. This ranked Ethiopia as the 55th largest meat-producing country in the world [31].

The reasons behind the low rate of meat processing in Ethiopia are multiple, including low offtake rates, low domestic consumption (8.5 kg/year), and low commercial bases of livestock production. Most producers sell their animals for cash needs, and bulls are culled from draught and cows from milk. The demand for input supply, particularly for improved animal genetic resources, has increased substantially with the poor response of the supply side. There is a gap in the coordination of efforts and in basing livestock development interventions on scientific knowledge with value chain in mind.

#### 2.3.1. Input Suppliers

Various services and inputs are supplied to the livestock sector in Ethiopia. Animal health, breed improvement, feed resource improvement, extension service and development, finance and market, research output, veterinary drugs, vaccines, machinery, equipment, and utensils, as well as knowledge [42]. Perhaps, the most provided service is veterinary service. The component and manner of the provision vary from place to place. Shortage of inputs in terms of appropriate technology unless it is augmented within puts which includes a shortage of improved animals, concentrate, and ingredients for balanced feeds, forage seeds, veterinary drugs, and equipment.

The demand for input supply of improved animal genetic resources has increased substantially despite the poor response from the supply side [25]. There is a gap in the coordination of efforts and in establishing the value chain. Therefore, in the future, livestock development interventions and scientific research need to shift the focus from predominantly developing new biophysical technologies toward social science research that assesses issues in the value chain, macroeconomic institutions, and policies that influence the adoption of technology [39].

There is a chronic shortage of trained manpower in the field of animal sciences. There is frequent movement of staff due to restructuring and the search for better job opportunities. Hence, this has had a negative impact on the development of livestock and meat quality. Human capacity development is a problem in meat-processing industries in Ethiopia [5, 7, 14]. To mitigate the shortage of human resources in meat-processing skills for both domestic and export markets, training should be developed in the short and long term. This training should be planned with collaborations of the Ethiopian Meat and Dairy Development Institute (EMDDI), universities, research institutes of science and technology, and technical and vocational and educational training (TVET) on generating better technologies for application in livestock production and product processing and allocation of adequate capital required on the supply side [39, 43, 44].

#### 2.3.2. Producers/Union/Cooperative Factors

Ethiopia is a largely rural country with an agrarian economy. Individual farmers or group fatteners and cooperatives are taken as livestock producers. Cattle farming has greatly contributed to the success of meat quality. Beef-producing cattle are normally reared extensively during their early stages of life and then sometimes transferred to intensive systems during the fattening and finishing stages [34, 45]. In Ethiopia, rural livestock production systems are subsistence oriented [36, 38].

The motivation for livestock sales is incidental household expenses (loan repayments, taxes, and social and family obligations) rather than preplanned commercial gain [4]. The procedure influencing to acknowledge certain meat or meat items is multidimensional. This indicates that many livestock keepers do not view their animals as commercial entities but rather as an asset that can be sold for cash when needed [36]. However, in the current scenario, meat quality demand is increasing from time to time at the expense of quantity [46].

However, according to the report [13, 27], the cattle availed for the market were too old because the producers did not primarily keep livestock for meat production, mostly females were culled for dairy purposes, and oxen ceased draught in poor body conditions. In recent times, a small fraction of Ethiopian beef raised in feedlots by smallholders throughout the country fattens, though the majority of cattle are raised in backyard systems [31, 32, 36].

Despite feedlots, cattle fatteners are perceived as producing higher-quality meat than backyard. Beef cattle producers normally rear cattle extensively during their early stages and then sometimes transfer to intensive systems during the finishing stages [34, 45]. Most cattle fed in the feedlot are 50–60-month-old Boran bulls targeted at a higher value for the export market rather than domestic ones [5, 34]. Generally, feedlot cattle fattening produces softer meat with white fat and a good proportion of red meat. This meat is preferred for fried steaks or *tibia* and *kurt*. Backyard-fattened meat is reported to be tougher, with yellow fat, more fat (but less marbling), and less red meat. This is preferred for consumption as raw meat for the local stew called *wot*. Backyard fattening is cheaper than feedlot operations but cannot supply large and consistent volumes to a commercial abattoir or trader [36].

#### 2.3.3. Traders

Traders any time buy animals and transport them to the district, terminal, and abroad market for profit margin. The perception of meat quality has been viewed differently by other stakeholders and from place to place, which is influenced by different factors [47, 48]. Formal trading is constrained by irregular and variable quality supplies, nonvalue added, late payment, and limited transparency on the health and weight condition of animals [5].

Nondedicated trucks and improper handlings like beating during collection and transportation are usually done by Ethiopian livestock traders. To increase marginal benefits, mixing animals of different ages, sexes, and novelty during transport might cause high numbers of animals to be stressed and killed by restraint [6, 12]. This chronic stress before slaughter leads to the depletion of stored glycogen, which results in dark and firm dry meat [41, 49]. Outlining guidelines of legal support, design of appropriate vehicles for use in transporting animals, and creating awareness on engaging in animal welfare are needed in a holistic approach for the trader.

#### 2.3.4. Middleman/Broker

Brokers are major actors in many livestock markets in Ethiopia. However, they are sometimes considered an unproductive (non-value-added actor) market chain, particularly in secondary and primary markets [5]. The engagement of long-chain actors has negatively affected producers and final meat consumers and hindered the effectiveness of the market. However, most central and abroad market brokers are major actors of livestock market in Ethiopia for mediation and transaction facilitation. They act as retailer demands and price negotiators between the buyer and seller via keeping commission from the sale of the animal on both sides. But, a large proportion of sales are on credit and incur late payment, limiting transparency in promotion of products on quality, health, and weight, which is vague for commodity satisfaction [5, 50].

#### 2.3.5. Abattoir Worker

Abattoir is a place where the process lives muscles are converted to meat. Hence, it needs careful handling, stunning, and postslaughter treatment; especially, temperature plays an important part in the final quality of the meat produced [48]. However, abattoirs in Ethiopia have inadequate facilities for processing [7, 15, 51, 52]. The poor facility, lack of workers' skills, and less sanitation of carcass transport are the main causes of the poor quality of meat [53]. The majority of Ethiopian slaughterhouses have no timely health checkups and training on the section. Most Ethiopian butchers get service from municipality abattoirs, who have fewer facilities than usual. Different abattoirs have different facilities and management systems that affect animal behavior at slaughter and the quality of the product indifferently [7]. Therefore, to increase the value chain of meat, Ethiopia needs to invest in modern abattoirs and meat-processing industries of international standard (HACCP certified) with all the necessary facilities to qualify for exporting processed and further processed meat products.

#### 2.3.6. Butchers/Restaurants

Butcher shops sell raw meat on a retail basis to consumers as well as roasted products [36, 37]. The butchers have preferred fattened animals for drip loss [32]. However, consumers prefer less-fat-content meat cuts due to perceiving high-fat-content meat as the cause of the health problem [54]. Otherwise, Ethiopian butcher men perceive female animals as a source of inferior meat quality. Hence, mostly female animals are slaughtered as part of a cultural ceremony and shared among the group called “*Kircha*” at the village level [55]. However, barren ewes are the most preferred type of sheep by butchers in some areas, such as Western Oromia and the Horro area. This is mainly related to their lower price and high meat yield as compared to young, growing ones in quality ways [50].

In contrast, the demand for meat is increasing not only in quantity but also quality-wise in the current scenario [46]. People with a higher social or economic status demand a greater amount of high-quality meat products [5, 27]. This contradictory perception is supported by the report in [56, 57], who report that the meat consumption pattern in Ethiopia is highly associated with the location, culture, and wealth status of consumers.

#### 2.3.7. Consumers

Meat is the main source of protein and has great physiological value for people [58]. Meat consumption patterns are unpredictable due to constant changes in consumer behavior towards meat and other food products [59]. For consumers to willingly purchase and consume a particular meat product, their perceptions of it must be positive [60]. Meat consumption keeps increasing every year around the world [61, 62]. However, Ethiopian meat consumption has ceased for years [10]. The per capita meat consumptions in Ethiopia are about 8.5 kg/year, which is the second-lowest in Africa [31]. There are several reasons for this low consumption, including low per capita income, high domestic meat prices, and the fasting days by the Orthodox Christians, which reduce aggregate demand by 20–35% [5].

Red meat and poultry utilization in Ethiopia have been associated with cultural practices [56]. Knowledge gained from experience, facts, stories, impressions, and the interests of an individual can influence perceptions of meat quality [1,2]. Consumers perceive the quality of meat to be associated with color, tenderness, juiciness, and leanness, combined in a unidimensional quality concept [2, 24]. Meat color is the first quality attribute that a consumer uses to predict freshness and wholesomeness [13, 56, 63]. The presentation of meat with the correct color is the most important aspect of the marketing of meat. Consumers tend to discriminate negatively against meat that is discolored [60]. Consumers can reject dark meat because it is perceived as coming from old or poorly handled animals and is described as being tough, having an undesirable flavor, and having a short shelf-life [64].

Most Ethiopian meat consumers are not satisfied without eating red meat (*Kurt*), even though they eat it cooked. However, negative effects with intermuscular fat in contrast to marbling are perceived as positively associated with visual quality. Reference [56] reports that raw meat ‘*Kurt*' is regarded as a cultural and social status indicator in the Wolaita zone of Ethiopia. The knowledge and good background of Ethiopian meat consumers on marbling are appreciated. Moreover, marbling affects the flavor, juiciness, and tenderness of the meat and increases its palatability [2]. Meat color has been positively related to a favorable evaluation of the expected visual quality of meat [3]. Therefore, the Ethiopian meat industry should have been fascinated by the knowledge of what quality cues consumers use when purchasing meat and how they can use this information to remain competitive. However, meat value chain actors in Ethiopia have a different perception of meat quality determinants. The summary is given in Table 1 [6].

Consumers from different countries and locations are evaluated on the quality of meat in different criteria to decide to repurchase. This is because different factors influence their preference when buying meat products [65, 66]. Different researchers from different countries, locations, and time periods reported consumer's different meat quality evaluation criteria to repurchase from butchers, supermarkets, or hotels (Table 2). Hence, it needs the contextual value chain signal as for consumer perceptions of meat quality for consumption and repurchasing.

### 2.4. Challenges and Opportunities of Live Animal and Meat Value Chain in Ethiopia

The opportunities and challenges of livestock and their products in Ethiopia in the future are faced with uncertainty. This is because, currently, decision-makers provide invaluable insights into actions. It needs a strong forward-looking approach when designing policies and investments in dynamic and rapidly changing societies [10].

#### 2.4.1. Challenge

Livestock product quantity and quality are paradoxical now in Ethiopia because the insights among meat value chain actors are not analogous. Establishing and jointly determining meat producers according to consumers' perceptions of quality determinants are an important theme of the future agenda in Ethiopia [10, 31].

Mostly, the livestock production system in Ethiopia is subsistence-oriented, male for traction and females for dairy purposes [36]. Therefore, mostly aged and unproductive animals were fetched to market and slaughterhouses with poor body conditions. Even though Ethiopia developed a beef cattle carcass classification system in 2012 GC, the system has not been used to characterize the carcass quality to date in all Ethiopian abattoirs [27].

In Ethiopia, the live animal and product value chain signals are challenged by many factors [5, 10, 31, 36, 56, 71]. Hence, the effort of the government in licensing each “actor” has a noticeably important role in creating a sustainable marketing environment where each actor can identify and adapt their competitive strategy in the value chain:

- The livestock production system is not market-oriented
- There is a lack of an integral connection between the actors involved in the production chain
- There is insufficient knowledge at different levels of actors
- There are prevalence of livestock diseases and inadequate veterinary support services
- There are inadequate infrastructures on transportation routes and markets
- Poor application of meat safety and hygienic protocols in domestic abattoirs and export markets is followed
- There is a lack of a quality-based meat pricing system to offer encouragement to producers
- There are inadequate research and extension programs in the production, processing, and marketing of meat
- Illegal trade is followed around the lowland borders of the country
- There is a lack of efficient air transport for the export of fresh and chilled meat
- Some markets are also dominated by influential actors/personalities
- There is insufficient use of technology for product processing and among value chains
- Poor facilities are observed, particularly at all chains

#### 2.4.2. Opportunities

The demand for live animals and meat in Ethiopia is a good opportunity for value chain actors in the future. High demand is due to growing populations, urbanization, economic growth, domestic consumption, official exports, and high demand for animals by the export abattoirs [38]. Recently, the government of Ethiopia recognised the importance of livestock in poverty alleviation and placed an emphasis on modernizing and commercializing the livestock subsector [4]. Therefore, Ethiopian live and animal product value chain actors have the following opportunity:

- There are increments in all livestock population species
- There is proximity to the Middle East market and South Africa
- Attention and support are given by the government for the sector
- Integrated agro-industrial parks are established in Ethiopia at strategic locations
- There is an increasing number of export abattoirs in Ethiopia with big investment opportunities
- There is the beginning of livestock registration and traceability systems at the pilot level
- The Livestock and Fishery Development Ministry in Ethiopia is coming into being
- There is global technology innovation

## 3. Conclusions and Recommendations

Ethiopian livestock marketing is characterized by poor market infrastructure, technical knowledge, and long-chain actors. The flow of the chain is less and there is biased communication among actors because they only perceive for their benefit rather than care for quality products in their hand for the next firm actors. This is because it is largely a personalized business with irregular buyers and sellers and steered by several brokers. Hence, more live animal exports are through unofficial channels; however, red meat exports pass through formal channels due to the high degree of regulation in importing countries. The lack of an effective grading system, absence of market information, absence of promotional, sustainable supply problems, transport problems, the prevalence of diseases, illegal export, inadequacy of infrastructure, competition, repeated bans, and inadequate port facilities are raised as challenges of the live animal and meat marketing system in Ethiopia.

Most of the butcher shops in Ethiopia hang meat in the open door, without protection from dust or flies. They do not refrigerate the meat overnight, not sterilize their containers after or before use, and wash it using soap/detergent powders and untreated water. Abattoir workers transport carcasses from the conveyer bar to the vehicle on their shoulders. No regular health checkups and training are given for abattoir workers. Meat consumption patterns in Ethiopia are unpredictable due to constant changes in consumer behavior towards meat and other food products associated with cultural and religious practices. The demand for live animals and meat in Ethiopia is a good opportunity for value chain actors in the future.

### 3.1. Recommendations

- Awareness should be created on strengthening alliances between actors to provide reliable and sustainable quality meat and meat products across market signals for both domestic and international markets
- Premiums should be paid to producers who bring young beef animals to market
- Governments should support and design to fill the bridge gap in meat value chain actor flows
- Safe meat must be maintained to ensure that public health is important through the implementation of GMP and HACCP principles
- Non-value-added actors from the livestock market (value-chain actors) should be limited
- Training should be provided to stakeholders on the economic importance of biosafety for animals
- Training and awareness should be done on welfare intimidation of slaughtered animals and how it affects meat quality
- Essential meat safety measures should be formulated to save product quality and consumers from food-borne infection and intoxication

## Figures and Tables

**Table 1:** Summary of some stakeholder perceptions on meat quality determinants as measured by severity.

Producers	Traders	Abattoir men	Butchers	Consumers
Feed resource	Beating	Stay in Lairiage	Health	Health
Health service	Feed resource	Breeding	Agroecology	Age of cattle
Breeds	Agroecology	Bleeding	Seasons	Seasons
Technologies	Age	Carcass handling	Age	Conformation
Fattening place	Breeds	Trekking/transport	Body condition	Cooking
Credit service	Transportation	Age	Stay in Lairage	Sex
Market	Sex	Loading and unloading	Sex	Cattle origin
Water supply	Meat cuts	Methods of stunning	Transport	Breeds
	Cooking	Agroecology	Cooking	
	Carcass transportation		Slaughtering	

Source: GADISA [6].

**Table 2:** Different consumers evaluating meat quality by different materials.

[13]	[56]	[6]	[36]	[67]	[64]	[65]	[66]	[68, 69]	[70]
Juisiness	Color	Color	Price	Tenderness	Ph	Appearance	Tenderness	Color	Price
Color	Fat level	Tenderness	Tenderness	Ph	Color	Origin	Taste	Fat content	Label
Tenderness	Taste	Leansess	Marbling	Color	Tenderness	price	Juiciness	Conivinent	Package
Flovor	Texture	Juicesness	Juiciness	Juiciness	Texture	Brand	Leaness	Promotion	Appearance
Leaness	Price	Flovor	Fattiness	Flavor	Juiciness		Safety	Cleaness	Trust
Shelf live		Smell	Color	Nutrative value			Convenience	Certification	Origin
Price		Price						Smeel
								Taste
								Tenderness
								Juiciness

## Data Availability

No data were used to support this study.

## References

[1] Michel F., Hartmann C., Siegrist M. (2021). Consumers’ associations, perceptions and acceptance of meat and plant-based meat alternatives. *Food Quality and Preference*.

[2] Miller R. (2020). Drivers of consumer liking for beef, pork, and lamb: a review. *Foods*.

[3] Vimiso P. (2012). Preliminary study on consumers’ and meat traders’ perceptions of beef quality and how the beef quality is affected by animal welfare practices. *Scientific Research and Essays*.

[4] Harko H. (2015). Review of beef cattle value chain in Ethiopia. *Industrial Engineering Letters*.

[5] EDMID (2016). *Ethiopian Agro-Industry Strategy Meat Industry Sub-Sector Strategic Plan*.

[6] Gadisa B. (2018). Analysis of eating qualities, perception of stakeholders on beef qualities determinants and commercial value of meat cuts in eastern Oromia, Ethiopia.

[7] Mummed Y. (2015). Beef carcass quality, yield and causes of condemnations in Ethiopia.

[8] Shapiro B. I., Gebru G., Solomon D., Negassa A., Kidus Negussie G. A., Mechal H. (2015). Ethiopia livestock master plan roadmaps for growth and transformation.

[9] Eshetie T., Kelifa H., Tadesse T. (2018). Meat production, consumption and marketing tradeoffs and potentials in Ethiopia and its effect on GDP growth: a review. *Journal of Nutritional Health & Food Engineering*.

[10] FAO (2019). The future of livestock in.

[11] Ngasala J. u. B., Nonga H. E., Mtambo M. M. A. (2015). Assessment of raw milk quality and stakeholders’ awareness on milk-borne health risks in Arusha City and Meru District, Tanzania. *Tropical Animal Health and Production*.

[12] Bulitta F. S., Gebresenbet G., Bosona T. (2012). Animal handling during supply for marketing and operations at an abattoir in developing country: the case of gudar market and ambo abattoir, Ethiopia. *Journal of Service Science and Management*.

[13] Gebeyehu A., Kolisch (2012). *Evaluation of Slaughter Parameters, Proximate Composition, Microbial Load and Eating Qualities of Ethiopian Beef of Arsi Cattle in Adama and Bishoftu Towns, Oromia, Ethiopia*.

[14] AACCSA (2015). *Value Chain Study on Meat Processing Industry in Ethiopia*.

[15] Zerabruk K., Retta N., Muleta D., Tefera A. T. (2019). Assessment of microbiological safety and quality of minced meat and meat contact surfaces in selected butcher shops of Addis Ababa, Ethiopia. *Journal of Food Quality*.

[16] Rani Z. T., Hugo A., Hugo C. J., Vimiso P., Muchenje V. (2017). Effect of post-slaughter handling during distribution on microbiological quality and safety of meat in the formal and informal sectors of South Africa: a review. *South African Journal of Animal Science*.

[17] Fromsa A., Jobre Y. (2012). Estimated annual economic loss from organ condemnation, decreased carcass weight and milk yield due to bovine hydatidosis (Echinococcus granulosus, Batsch, 1786) in Ethiopia. *Ethiopian Veterinary Journal*.

[18] FAO (2005). The state of food and agriculture: agricultural trade and poverty. *FAO Agriculture Series*.

[19] Asmamaw Aki Jano (2018). Survey on luna export slaughter house in eastern showa administrative zone of Oromia, Ethiopia. *Report and Opinion*.

[20] Gebru G., Gebretinsae T. (2018). Evaluating the implementation of hazard analysis critical control point (HACCP) in small scale Abattoirs of Tigray region, Ethiopia. *Food Protection Trends*.

[21] Leksmono C., Young J., Hooton N., Muriuki H. (2006). *Informal Traders Lock Horns with the Formal Milk Industry: The Role of Research in Pro-Poor Dairy Policy Shift in Kenya*.

[22] Grace D. (2014). The business case for one health. *Onderstepoort Journal of Veterinary Research*.

[23] Onono J. O., Wieland B., Rushton J. (2013). Factors influencing choice of veterinary service provider by pastoralist in Kenya. *Tropical Animal Health and Production*.

[24] Chulayo A. Y., Tada O., Muchenje V. (2012). Research on pre-slaughter stress and meat quality: a review of challenges faced under practical conditions. *Applied Animal Husbandry & Rural Development*.

[25] Assefa A., Hailu A., Mustefa A. (2021). Characterization, conservation and sustainable utilization of Ethiopian animal genetic resources: status, challenges and opportunities: a review. *International Journal of Social Science Studies*.

[26] LMD (2013). Agricultural growth program-livestock market development end market analysis for meat/live animals, leather and leather products, dairy products value chains expanding livestock markets for the small-holder producers.

[27] YesihakYusuf M., Edward C. W. (2015). Carcass quality audit-a strategy to improve beef sector in Ethiopia. *African Journal of Agricultural Research*.

[28] Ashenafi M., Addisu J., Shimelis M., Legese G. (2013). *Analysis of Sheep Value Chains in Doyogena, Southern Ethiopia*.

[29] Tigabie A., Lemma M., Erchafo T. (2021). *Community Conversations on Collective Livestock Marketing: The Case of Doyogena*.

[30] Anteneh B., Tegegne A., Beyene F., Gebremedhin B. (2010). Cattle milk and meat production and marketing systems and opportunities for market orientation in Fogera Woreda, Amhara region, Ethiopia. *Improving Productivity and Market Success of Ethiopian Farmers Project Working paper 19*.

[31] Brasesco F., Asgedom D., Sommacal V. (2019). *Strategic Analysis and Intervention Plan for Live Animals and Red Meat in the Agro-Commodities Procurement Zone of the Pilot Integrated Agro-Industrial Park in Central-Eastern Oromia, Ethiopia*.

[32] Sintayehu G., Samuel Amare, Baker D., Solomon A., Ryan D. (2013). *Study of the Ethiopian Live Cattle and Beef Value Chain*.

[33] Alemayehu Y., Adicha A., Mengistu M., Eshetu B. (2016). Assessments of market oriented beef cattle fattening system under farmer management condition in South Omo zone of snnpr. *Current Research in Agricultural Sciences*.

[34] Dadi G., Teklebrhan T. (2017). Assessment of commercial beef cattle fattening practices and performance in East shoa zone. *International Journal of Agricultural Science and Food Technology*.

[35] Ameha S. (2011). Export requirements for meat and live small ruminants how can development agents assist producers to improve small ruminant export.

[36] Yami A., Gelaw F., Koster H., Siraw B. (2018). Reorienting livestock production to respond to the meat quality requirements of high-end domestic and export markets.

[37] Tesfaye M. (2016). Assessment of beef cattle production, management practices and marketing system in lume distr district of east Shoa zone, Ethiopia Msc thesis tesfaye moreda tessema college of agriculture.

[38] Eshetu E., Abraham Z. (2016). Review on live animal and meat export marketing system in Ethiopia: challenges and opportunities. *Journal of Scientific and Innovative Research*.

[39] Kebebe E. (2015). Understanding factors affecting technology adoption in smallholder livestock production systems in Ethiopia: the role of farm resources and the enabling environment. https://library.wur.nl/WebQuery/wda/2092836.

[40] Diamond A., Tropp D., James Barham, Frain Muldoon M., Kiraly S. (2014). *Non-food Value Chains*.

[41] Birhanu A. F., Mummed Y. Y., Kurtu M. Y., O’Quinn T., Jiru Y. T. (2019). Level of pre-slaughter stress and quality of beef from Arsi, Boran and Harar cattle breeds in Ethiopia. *Cogent Food & Agriculture*.

[42] ATO (2020). Proceedings of drivers of agricultural transformation in Oromia.

[43] Asmare B. (2022). A review of sensor technologies applicable for domestic livestock production and health management. *Advances in Agriculture*.

[44] Zemedu A. A. L. (2015). Contribution of livestock sector in Ethiopian economy: a review. *Advances in Life Science and Technology*.

[45] Probst J. K., Hillmann E., Leiber F., Kreuzer M., Spengler Neff A. (2013). Influence of gentle touching applied few weeks before slaughter on avoidance distance and slaughter stress in finishing cattle. *Applied Animal Behaviour Science*.

[46] Tefera T. D., Mummed Y. Y., Kurtu M. Y., Letta M. U., O’Quine T. G., Vipham J. L. (2019). Effect of age and breeds of cattle on carcass and meat characteristics of Arsi, boran, and harar cattle in Ethiopia. *Open Journal of Animal Sciences*.

[47] Geletu U. S., Usmael M. A., Mummed Y. Y., Ibrahim A. M. (2021). Quality of cattle meat and its compositional constituents. *Veterinary Medicine International*.

[48] Kiran M., Nithin Prabhu K., Paramesha S. C. (2018). Consumption pattern, consumer attitude and consumer perception on meat quality and safety in Southern India. *International Food Research Journal*.

[49] Adzitey F., Nurul H. (2011). Pale soft exudative (PSE) and dark firm dry (DFD) meats: causes and measures to reduce these incidences - a mini review. *International Food Research Journal*.

[50] Legese G., Fadiga M. (2014). *Small Ruminant Value Chain Development in Ethiopia: Situation Analysis and Trends*.

[51] Birmaduma G., Yesihak Y., Mohammad Y. K. (2019). Evaluation of physical facilities, operation and management practice in selective public abattoirs in eastern Oromia, Ethiopia. *International Journal of Agricultural Science and Food Technology*.

[52] Gebeyehu A. (2013). Evaluation of microbial load of beef of Arsi cattle in Adama town, Oromia, Ethiopia. *Journal of Food Processing & Technology*.

[53] Ismail S. A. (2006). Microbiological quality of hawawshy consumed in Ismailia, Egypt. *Journal of Food Safety*.

[54] Gadisa B., Yusuf Y., Kurtu M. Y. (2019). Assessment of butchers and consumers perceptions on beef quality determinants in Adama and Dire Dawa towns, Ethiopia.

[55] Seleshe S., Jo C., Lee M. (2014). Meat consumption culture in Ethiopia. *Korean Journal for Food Science of Animal Resources*.

[56] Amistu K., Ermias B., Asrat A. (2017). Consumer Preference of raw beef (“kurt”) in Wolaita sodo town , Southern Ethiopia. *Journal of Food and Dairy Technology*.

[57] Xazela N. M., Hugo A., Marume U., Muchenje V. (2017). Perceptions of rural consumers on the aspects of meat quality and health implications associated with meat consumption. *Sustainability*.

[58] Shi Y., Wang X., Borhan M. S. (2021). A review on meat quality evaluation methods based on non-destructive computer vision and artificial intelligence technologies. *Food Science of Animal Resources*.

[59] Escriba-Perez C., Baviera-Puig A., Buitrago-Vera J., Montero-Vicente L. (2017). Consumer profile analysis for different types of meat in Spain. *Meat Science*.

[60] Troy D. J., Kerry J. P. (2010). Consumer perception and the role of science in the meat industry. *Meat Science*.

[61] De Smet S., Vossen E. (2016). Meat: the balance between nutrition and health. A review. *Meat Science*.

[62] Stephens N., Di Silvio L., Dunsford I., Ellis M., Glencross A., Sexton A. (2018). Bringing cultured meat to market: technical, socio-political, and regulatory challenges in cellular agriculture. *Trends in Food Science & Technology*.

[63] Jildau v. B. (2014). Consumers ‘quality perception of steak from the supermarket vs . the in-store butcher.

[64] Ponnampalam E. N., Hopkins D. L., Bruce H., Li D., Baldi G., Bekhit A. E. (2017). Causes and contributing factors to “dark cutting” meat: current trends and future directions: a review. *Comprehensive Reviews in Food Science and Food Safety*.

[65] Svetlíková V., Palkovič J., Poláková Z., Fusková M. (2018). Factors affecting consumer behaviour in case of meat with an emphasis on the price. *International Scientific Days 2018. Towards Productive, Sustainable and Resilient Global Agriculture and Food Systems: Proceedings*.

[66] Webb E. C. (2006). Manipulating beef quality through feeding. *South African Journal of Food Science Nutrition*.

[67] Webb E. C., Erasmus L. J. (2014). The effect of production system and management practices on the quality of meat products from ruminant livestock. *South African Journal of Animal Science*.

[68] Font-i-Furnols M., Guerrero L. (2014). Consumer preference, behavior and perception about meat and meat products: an overview. *Meat Science*.

[69] Vermeulen H., Schönfeldt H. C., Pretorius B. (2015). A consumer perspective of the SA red meat classification system. *South African Journal of Animal Science*.

[70] Bytyqi N., Verçuni A., Pllana M., Jahja A., Bytyqi H. (2012). Analysis of consumer behavior in regard to the beef meat in kosovo. *Food and Nutrition Sciences*.

[71] Tena Y. T., Asgedom A. H., Gebre Y. T. (2015). Sheep and goat marketing and consumption in relation to religious festivities in shifting and permanent farming systems in Western Ethiopia. *Global Journal of Animal Scientific Research*.

